# Analysis and Experimental Investigation of the Light Dimming Effect on Automotive Visible Light Communications Performances

**DOI:** 10.3390/s21134446

**Published:** 2021-06-29

**Authors:** Cătălin Beguni, Alin-Mihai Căilean, Sebastian-Andrei Avătămăniței, Mihai Dimian

**Affiliations:** 1 Integrated Center for Research, Development and Innovation in Advanced Materials, Nanotechnologies, and Distributed Systems for Fabrication and Control, Stefan cel Mare University of Suceava, 720229 Suceava, Romania; catalin.beguni@usm.ro (C.B.); sebastian.avatamanitei@usm.ro (S.-A.A.); dimian@usm.ro (M.D.); 2 Department of Computers, Electronics and Automation, Stefan cel Mare University of Suceava, 720229 Suceava, Romania

**Keywords:** inter-vehicle communications, light dimming, “lights-off” visible light communications, optical communications, vehicle-to-vehicle communications, vehicular communications, visible light communication

## Abstract

The use of Visible Light Communications (VLC) in vehicular applications has become a major research area due to its simplicity, high performance to cost ratio, and great deployment potential. In this context, this article provides one of the very few analyses and experimental evaluations concerning the integration of a light dimming function in vehicular VLC systems. For this purpose, a vehicle-to-vehicle VLC prototype has been implemented and used to evaluate the systems’ communication performances in light dimming conditions, while decreasing the duty cycle from 40% to 1%, and increasing the communication range from 1 to 40–50 m. The experimental results showed that in normal lighting conditions, the VLC technology can easily support low duty cycle light dimming for ranges up to 40 m, while maintaining a 10^−6^ BER. Nevertheless, in strong optical noise conditions, when the system reaches its SNR limit, the communication range can decrease by half, whereas the BER can increase by 2–4 orders of magnitude. This article provides consistent evidence concerning the high potential of the VLC technology to support inter-vehicle communication links, even in light dimming conditions.

## 1. Introduction

Mobility is fundamental for most human activities; thus, the safety of the transportation system is a stringent research topic for the automotive industry, for vehicle manufacturers, and for the academic community. Preoccupation with this aspect has been an important subject for more than a century of automotive history, not only through controlling driver behavior, but also through improvements made in the design of vehicles and road infrastructures. Nowadays, intelligent systems implemented in cars and transport infrastructures are widespread, providing innovative services in terms of safety [1]. Increasing concern in this area has led to the implementation of new concepts, which incorporate state-of-the-art wireless communications technologies that enable smart vehicles to share information with other vehicles and with the transportation network, in order to limit the risk of accidents [2,3,4,5] and to improve the efficiency of road transportation.

**Figure 1:**
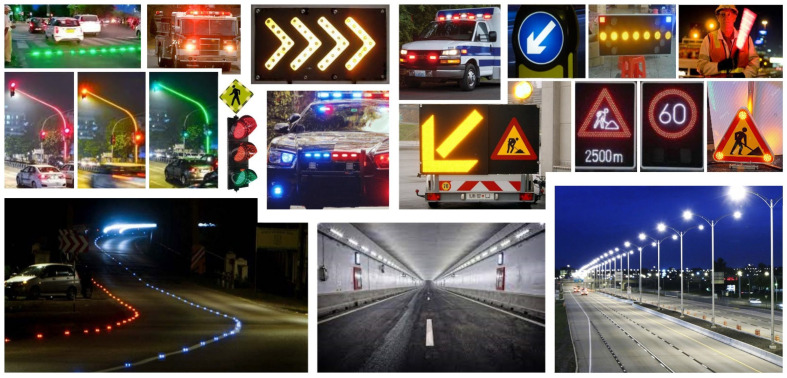
LED-based lighting solutions integrated in vehicles and in road transportation infrastructures. This wide diffusion facilitates the deployment of the VLC technology.

Being superior to incandescent bulbs and fluorescent tubes in terms of efficiency, life term expectancy, and high tolerance to humidity, LEDs are on the way to becoming the new normal in lighting [6,7]. Thus, their market share is gradually increasing, as LEDs are replacing other lighting solutions not only in home settings but also in vehicle lighting systems and in transportation lighting infrastructures (see Figure 1). Furthermore, due to their fast-switching ability, LEDs also enable the usage of the visible light spectrum for data transmission, in addition to their main lighting or signaling function. 

In short, Visible Light Communications (VLC) technology involves a VLC emitter that has a lighting function and a VLC receiver. In order to enable the information transfer, the data to send are modulated onto the VLC emitter’s optical carrier at frequencies that are unperceivable by the human eye. The VLC receiver converts the modulated light into an electrical signal, from which data can be extracted by various processing techniques. Thus, the VLC technology can turn virtually any LED light source into a data transmission device, standing out as one of the possible solutions in communication-based vehicle safety applications [1,4,5]. In this area, the performances of automotive VLC systems have continuously improved [4,5,6,7,8,9,10,11], although there are still challenges that require further attention [5]. Therefore, if a vehicle has to deal with an unexpected event, various data, including inter-vehicle distance [12,13,14], can be passed to the rear vehicles, in order to avoid an accident or a traffic jam. In road transport applications, there are currently many LED light sources as part of the road infrastructure and of the vehicle lighting systems, so VLC technology is easier to implement [5], while providing many advantages, such as low latencies [10,11], and being complementarity to 802.11p RF-based technology [15], where the VLC technology can counteract most common vulnerabilities associated with RF interferences in heavy traffic scenarios.

Dissimilar to any other wireless communication technology, in VLC, the data carrier is visible to the human eye. Under these circumstances, the IEEE 802.15.7 standard for short-range optical communications [16,17] introduces strict requirements, according to which the use of a light source in data transmission applications must not affect in any way the main function of the device. As the lighting function is considered of a higher priority compared to the data transmission function, several obstacles emerge, whereas technical solutions have already been found [11,17,18]. One of the conditions the standard imposes is related to light dimming. Thus, the VLC emitter must be able to support light dimming while providing optical wireless data transfer. If it is to apply this regulation to vehicular applications, one can understand that light dimming is less stringent, whereas the use cases that could integrate light dimming are less numerous. Nevertheless, in the context of applications for energy harvesting technologies gaining popularity, several possibilities emerge. Thus, light dimming could be used in street lighting applications, informative panels, or advertising displays. In such scenarios, the light source optical power could be adapted in accordance with ambient light, whereas in the case of battery-operated devices, the output light intensity could be corroborated with the remaining available power, including the special case of electric vehicles. This function would be very suitable in auto-dimmable headlights, enabling the vehicle to change the light intensity in order to prevent drivers from sudden glare at night conditions. In this context, the existing literature focused on the usage of the VLC technology in automotive applications provides very few investigations concerning the effect of light dimming on the performances of vehicular VLC applications. On the other hand, these effects have been only partially investigated in indoor applications, pointing out that further investigations concerning the effect of light dimming on the VLC performances are required.

In this context, the present article provides an analysis and an intensive experimental investigation concerning the effect of light dimming on automotive VLC performances. This article considers a Vehicle-to-Vehicle (V2V) communication scenario, analyzing the case when the brake lights transmit the data, but the intensity of the modulated light is dimmed to a level that can be considered as taillights by the current regulations. In order to provide a comprehensive investigation, the V2V VLC system operating in light dimming mode is tested in controlled laboratory conditions as well as in outdoor daytime uncontrolled conditions. The experimental evaluation is performed for variable communication distances, different lighting conditions, and variable duty cycles. Additionally, the particular case of dimming the light to such an extent that can be considered “off” for the human eye, but the transmission of data is still going on with ultra-short pulses, is analyzed. As far as we know, this article is one of the very few works addressing the issues associated with light dimming in vehicular VLC links. As this topic has been neglected in the existing literature addressing automotive VLC applications, this work provides important evidence that demonstrates the ability of the VLC technology to be compatible with such use scenarios. The rest of this article is structured as follows. Section 2 and Section 3 provide an overview concerning the issues associated with light dimming in VLC applications and on the existing solutions to these problems. Section 4 presents the V2V VLC prototype that has been used in the experimental evaluations. Section 5 describes the experimental evaluation procedure and presents the experimental results, whereas Section 6 provides a discussion concerning these results. Finally, Section 7 provides the conclusions of this work.

## 2. State-of-the-Art in Light Dimming and Lights-Off Visible Light Communications

### 2.1. Light Dimming in IEEE 802.15.7 Standard for Optical Communications

**Figure 2:**
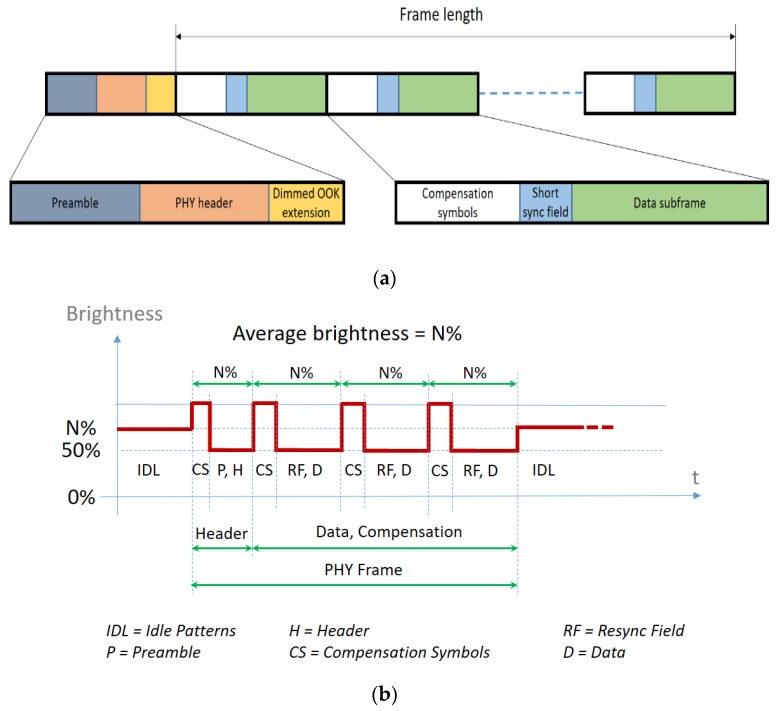

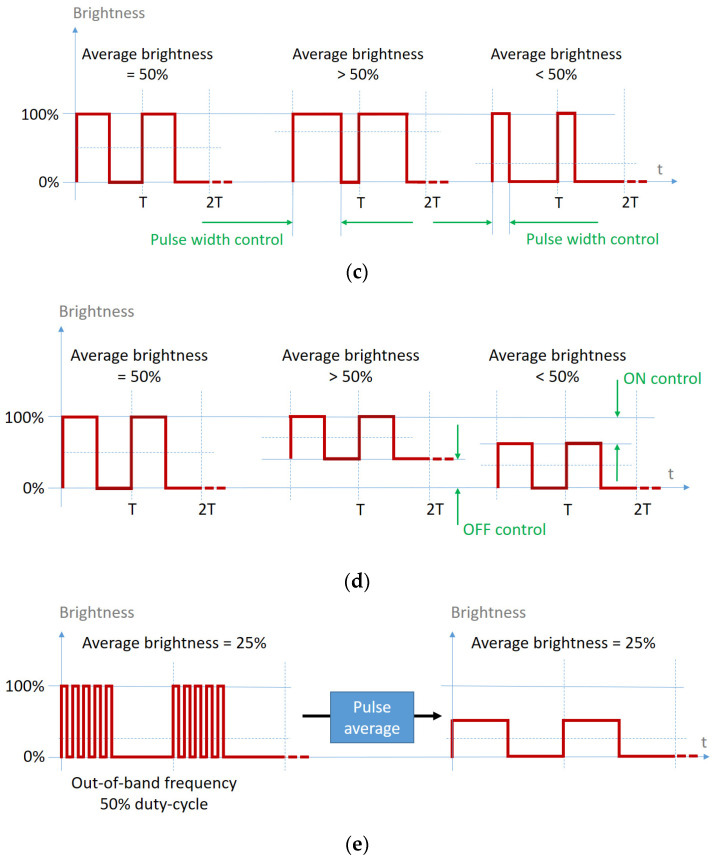
Light dimming in VLC: (**a**) The frame structure for the method of inserting compensation symbols; (**b**) Example of controlling the brightness in OOK modulation by adding compensation symbols; (**c**) Example of controlling the brightness in OOK modulation by pulse width variation; (**d**) Example of varying the brightness by controlling the amplitude of ones and/or zeros; (**e**) Example of varying the brightness in the out-of-band frequency.

One of the many obstacles in the development of the Visible Light Communications technology is standardization [17,19,20,21], so different attempts to standardize this technology have been made. Among the different standards that have been proposed, the IEEE 802.15.7 [16] seems to be the one that has the highest complexity and the widest acceptance. This standard has been developed with various challenges in mind, such as avoidance of flickering, dimming support, and maintaining communication when the lights are necessary to be off. As debated in [17], although the standard has drawbacks and vulnerabilities, considering the importance of standardization, the IEEE 802.15.7 standard and its revisions remain for the moment the most covering solution for VLC applications. The lights-off communication can be seen as a particular case of communication in dim conditions, but with an average brightness so low, that it cannot be perceived by the human eye, and therefore, the LED is considered to be “off.” The IEEE 802.15.7 standard specifies various methods for dimming support during data transmission, but also during idle periods when no data are being transmitted. Inserting compensation symbols (i.e., idle patterns and compensation time), controlling pulse width, adjusting the amplitude of the signal, and/or controlling in the out-of-band frequency (including the option of using the particular case of an un-modulated DC bias) are the major techniques proposed for dimming support [16,17,18]. Figure 2 illustrates the light dimming mechanisms for these techniques, which can simultaneously be used for flicker mitigation.

Broadly speaking, there are two main techniques for dimming the lighting of an LED source: analog and digital. The analog dimming technique is based on the Continuous Current Reduction (CCR), where the luminous flux is decreased by directly diminishing the forward current through an LED. This type of dimming control can be used with various modulations, such as Pulse-Amplitude Modulation (PAM). The main issue of this technique is the chromaticity shift of the light, due to the under-voltage applied to the LED [22]. Although it has been established that the use of digital dimming is recommended to avoid this issue, a relatively recent study [23] showed that color deviation makes its presence felt even in the digital case, due to different junction temperatures at various fill factor values.

The digital dimming techniques can be applied to many modulation methods for VLC data transmission, such as Pulse Position Modulation (PPM). The modulated VLC data signal is employed over a digital control signal used in a dimming technique, such as Pulse-Width Modulation (PWM). On these grounds, the IEEE 802.15.7 standard has introduced the Variable Pulse Position Modulation (VPPM), which combines the ability of PWM to provide simple light dimming with the data encoding mechanism of PPM. The resulting VPPM technique is able to provide high-resolution dimming and flicker-free communication while providing constant data rates.

### 2.2. Related Work

**Figure 3:**
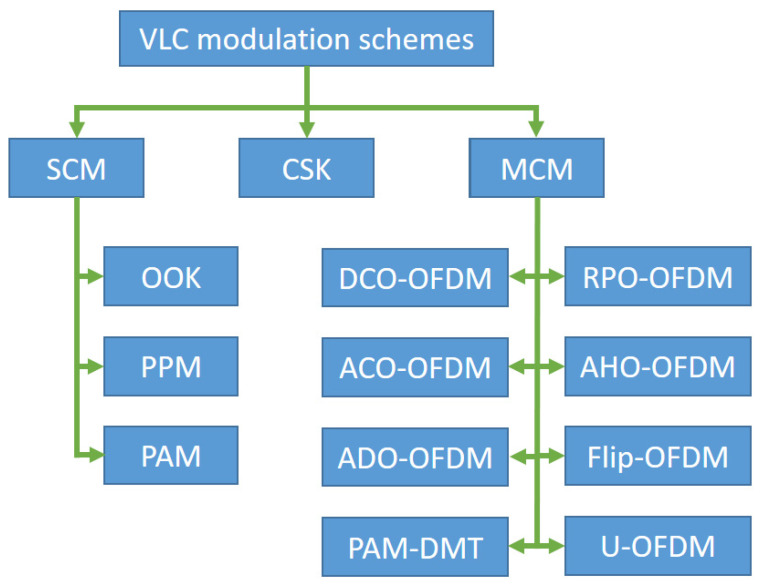
VLC modulation schemes.

The data transmission in VLC is done through Intensity Modulation (IM), i.e., the variation of the intensity of light in the time domain, and the data reception is done through Direct Detection (DD). The capability of an LED in terms of bandwidth is no more than a few megahertz, so one research area is related to modulation formats that can overcome this limitation. Based on this requirement, the modulation techniques mainly used in VLC can be classified as Single Carrier Modulation (SCM), Color Shift Keying (CSK) modulation, and Multi-Carrier Modulation (MCM) [24] (Figure 3). Various dimming techniques can be implemented based on these modulations.

#### 2.2.1. Single Carrier Modulations

The most common single-carrier modulations used in VLC are On-Off Keying (OOK), Pulse Position Modulation (PPM), and Pulse Amplitude Modulation (PAM).

Some of the most important advantages of the OOK modulation are the low cost and simplicity. OOK is the simplest Amplitude Shift Keying (ASK) modulation method. Usually, it has a square signal, and the amplitude is on two levels: high power and low power. OOK can use different coding formats, such as Non-Return to Zero (NRZ), Return to Zero (RZ), or Manchester coding. The RZ code ensures the returning to a low power level even when a series of “1” bits are needed to be transmitted. The big advantage here is the easier recovery of the clock from the data signal, with the exception of long series of “0” bits, a situation where Manchester code is more suitable.

In order to have dimming capability, one possible approach is Variable On-Off Keying (VOOK), through the insertion of compensation time [18]. In [25], the authors argue that in order to be perceived as “off,” the illumination limit of an LED device in indoor scenarios is dependent on the ambient light, which means that in broad daylight the VLC emitter can communicate with an increased level of power, without affecting the lights-off perception in the room. One drawback, however, is the fact that in vehicle scenarios, the LED emitter is in many cases directly observed, so the glowing must be much lower than in the indirect observance.

PPM is an attractive modulation technique for optical communications, offering an increased efficiency in power transmission. There are different types of PPM that can support dimming control, such as VPPM, and Multiple Pulse Position Modulation (MPPM) with its variants: Overlapping Pulse Position Modulation (OPPM), Differential Amplitude Pulse Position Modulation (DAPPM), Digital Pulse-Interval Modulation (DPIM), and so on.

Although is easy to implement the VPPM scheme, which is a combination of PWM brightness control with a 2-PPM modulation for communication proposed by the IEEE 802.15.7 standard group, the data rate limitation imposed by the binary implementation asks for a better method of digital dimming. One study [26] evaluated the performance of an *M-ary* VPPM scheme with dimmable capability for VLC, with increased performance of achievable data rate being demonstrated for a large modulation order. In order to counteract the rapid brightness fluctuations, a novel VPPM scheme was proposed in [27], where the targeted dimming level is achieved based on a step-by-step change in the brightness of the LED while moving average correlation masks are used to keep up with the change.

Controlling the number of pulses in each signal block, an MPPM communication with dimming support was proposed in [28], the authors arguing that this modulation is more efficient in terms of power and spectrum requirements than VOOK or VPPM. These results confirm the potential of this type of modulation, as seen in other studies, such as [29], where the band-utilization improved efficiency of MPPM over the PPM was demonstrated. As opposed to classical solutions, where two different modulations are used when dimming is necessary, a Variable-Rate Multi-Pulse Position Modulation (VR-MPPM) was proposed in order to jointly control the brightness control and data transmission in a VLC system based on white LEDs [30]. A novel dimming control scheme for VLC systems was proposed in [31]. Based on MPPM, the system can keep an average light intensity to attain different dimming targets. Another design was made in [32] to provide “lights-off” communication at 1.6-kbps data rate, over a distance of 1.3 m, using OPPM (encoding 10 times more bits per light pulse than OOK). Here, there were three challenges to overcome: to generate and detect ultra-short pulses of light with off-the-shelf LEDs and photodiodes, to find a suitable modulation and demodulation design, and to solve the collision errors when pulses from different transmitters are received. The authors even conducted a study to systematically examine user’s perception of the ultrashort light pulses. There was a study based on PPM [33] on the possibilities to transmit data in a smart room when the lights appear off, on the minimum illuminance needed for receiving the information, and on the factors that influence Signal-to-Noise Ratio (SNR) and Bit Error Ratio (BER), selecting the parameters and the requirements needed in terms of power, bandwidth efficiency, and dimming factor.

In [34], the desired dimming control was achieved by optimizing the probability and the intensity of the PAM constellation points with a hybrid scheme that combines the analog and the digital technique. Another system was proposed in [35], where a multilevel transmission scheme has the dimming controlled through the concatenation of PAM symbols, in order to obtain an average amplitude in line with the dimming requirement.

#### 2.2.2. Color Shift Keying Modulation

Two types of techniques are typically used for dimming control in a CSK environment: PAM and PWM. In [36], the CSK modulation positions the signal constellation based on colors, whereas the brightness is controlled through the optical power adjustments of the RGB LED. Color Intensity Modulation (CIM) is developed in [37] using multicolored LEDs, to allow simultaneously color matching and dimming requirements. Recently, a combined CSK-PPM modulation has drawn a lot of attention, with a PWM dimming control implemented in a similar way as for VPPM [38].

#### 2.2.3. Multi-Carrier Modulation

Orthogonal Frequency Division Multiplexing (OFDM) is a special class of MCM system that has attracted attention in the optical communication field as a bandwidth-efficient multiplexing transmission.

The two features related to multicarrier modulation are giving OFDM some advantages over other types of modulations: the ability to divide the OFDM spectrum into multiple sub-bands, which eases the design, and the adaptation of pilot subcarriers simultaneously with data carriers, offering a convenient possibility for phase and channel estimation [39]. An adaptive PWM dimming control scheme was used in [40] to achieve a BER of less than 10^−3^ in a variable Multi-level Quadrature Amplitude Modulation (M-QAM) OFDM VLC system, where a number of points in each constellation M are adapted in direct relation with the duty cycle at first, followed by the change of the symbol rate. The strong point of this approach is that the required power to drive the LED lamp is much less than in the case of an OOK modulation.

#### 2.2.4. Coding Techniques

There are two types of coding in VLC systems: line coding and Forward Error Correction (FEC) codes, such as Reed–Solomon codes. Technical design considerations for improving the physical layer of VLC systems by reviewing the latest developments in modulation and coding technologies were presented in [41]. The main technical challenges for improving the VLC physical layer under lighting limitations are also described.

## 3. Factors That Could Affect the Performances of Dimming in Visible Light Communications

As described in Section 2, several solutions to enable VLC in light dimming conditions have been proposed. Nevertheless, some initial conclusions can be drawn from these studies, as starting points for some guidelines. First of all, there is no intensive study (to the best of our knowledge) that analyzes the dimming and/or “lights-off” visible light communication in the automotive field. The overwhelming majority of these papers focused on indoor applications. Moreover, most of the existing concepts have only been evaluated analytically or by simulation means. However, these studies can be very useful in researching the applicability in the vehicular field, even if the practical implementation of such VLC solutions is rather challenging. For example, some of these techniques lead to other situations that are very difficult to handle, whereas in other cases, these lead to a very complex signal processing approach. Additionally, the use of VPPM to enable light variable dimming between 1% and 50% generates a significant variation of the pulse width, which significantly complicates the signal filtering process. Thus, the brightness dimming of a pulse train of a certain frequency leads to a proportional decrease in the width of each of its pulses (see Figure 2), and in the end, it is similar to a message transmitted using a higher frequency. Consequently, such a pulse will be affected by a low-pass filter having a cut-off frequency calculated based on the initial frequency. Thus, if we have a 400 kHz message, and apply a 1% dimming, the positive pulse width decreases from 1.25 µs to 25 ns. In such a case, the limit of the low-pass filter should be adapted as well. A possible solution to this problem would be to use an adaptive filter, of which the cut-off frequency is modified as a function of the dimming percentage. Nevertheless, as the standard for light dimming imposes a 0.1% resolution, the hardware complexity of an optimally matched analog filter significantly increases due to the high number of possible pulse widths. In such a case, the size of the circuit and the cost of the device significantly increase. A possible solution to this new problem would be the use of variable value components (i.e., digitally-programmable potentiometers, such as MCP41100) that would enable software control of the band-pass frequency. Nevertheless, in many cases, such components are not compatible with high-frequency applications. A different solution would be to use a wider band-pass filter. This approach has the advantage of a simpler hardware configuration that will allow the passage of a wider frequency range. On the downside, as the bandwidth increases, the additional noise components will be allowed to pass, affecting, in turn, the SNR of the received data signal.

This light dimming effect on the pulse width generates additional hardware problems. From the VLC emitter perspective, the narrow pulses involve LED drivers with a faster response, whereas in some cases, the light dimming performances could be limited by the LED’s characteristics. It is well-known that LEDs have switching times that can go down to a few nanoseconds. Nevertheless, the LED’s switching times are influenced by their capacitance. Thus, high-power LEDs, such as the ones used in vehicle lighting systems or in street lighting systems, have higher surfaces and in turn higher capacitances, affecting significantly the switching times, which can be hundreds of times higher. In such conditions, it is highly possible that most of the high-power LEDs will not be able to sustain a reliable link communication for light dimming levels below certain limits. The problems caused by light dimming become more stringent at the VLC receiver level. In this case, the VLC receiver should be designed with components that have lower switching times, with operational amplifiers that have higher slew rates, with DSP-based architecture that have significantly higher sampling frequencies, and with considerably more performant data processing units. All these requirements will increase the overall cost and/or could affect the reliability of the VLC system as a whole.

Being designed for VLC applications with low data rate requirements in outdoor conditions, the physical layer type I (PHY I) operating mode described in the IEEE 802.15.7 standard is the appropriate choice for vehicular VLC applications (Table 1). For such applications, the IEEE 802.15.7 standard specifies the use of OOK modulation with Manchester coding at data rates between 11.67 and 100 kb/s. Additionally, the standard also mentions the use of VPPM and of 4B6B coding providing data rates between 35.56 and 266.6 kb/s. In this case, the light dimming is achieved by modifying the duty cycle, whereas in the OOK case, this function is achieved by the insertion of compensation symbols and/or idle patterns (see Figure 2b). The insertion of compensation symbols and idle patterns accomplishes the light dimming at the cost of the data rate but ensuring at the same time a constant range. In such a case, the total duration of the compensation symbols or idle times is established based on the dimming percent. Thus, as the dimming percent decreases, the entire duration with no data transmission is increasing.

Now, if one is to apply this approach to the PHY I OOK use case, it is clear that this will lead to extremely low data rates that can hamper the safety concept, which should be the main function of a communication-based vehicle application. Consequently, this approach can be considered unsuitable for such applications, pointing out that the use of the VPPM technique for brightness control is more adequate for the automotive industry.

In order to achieve a dimming resolution of 0.1%, an algorithm is described in the IEEE 802.15.7 standard, the definition for VPPM mode being illustrated in Table 2. Because the VPPM affects the illumination of the transmitter LED, the communication performances must be expressed as a function of the distance, dimming ratio, and environment settings in a given VLC scenario [42]. The avoidance of flickering is mandatory in an automotive VLC system, so extra care is needed in this regard.

## 4. Description of the Visible Light Communications System

In order to experimentally evaluate the effect of light dimming on vehicular VLC performances, a VLC prototype has been implemented. The proposed architecture has been described in [43], where the VLC prototype has been used to evaluate the influence of vehicle misalignment over the performances of V2V communications. Thus, this section will provide a brief description that offers an adequate understanding of the concept, while also focusing on the improvements needed to enable light dimming.

**Figure 4:**
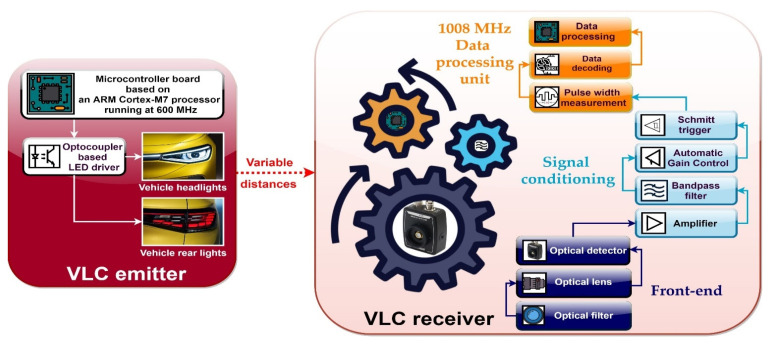
Schematic representation of the VLC system.

**Figure 5:**
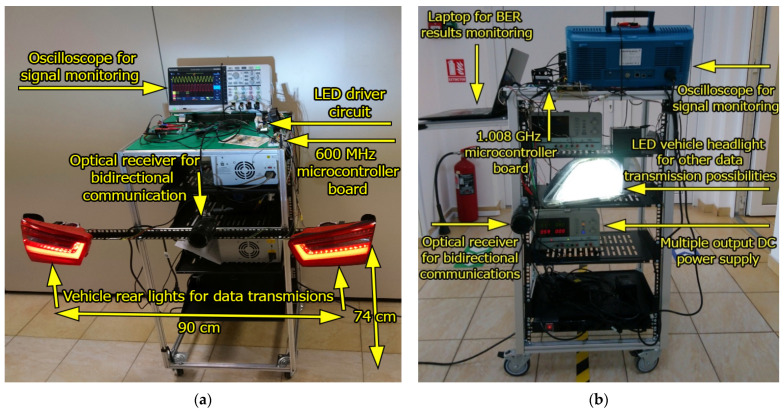
Hardware implementation of the visible light communication prototype for light dimming: (**a**) Emitter; (**b**) Receiver.

The schematic of the proposed VLC system is illustrated in Figure 4, whereas its hardware implementation is shown in Figure 5. The most important upgrades consist of the use of a more performant data processing unit and a more accurate data decoding process, which make possible the dimming function.

### 4.1. Discussion of the VLC Emitter Structure

The proposed VLC emitter is developed based on a set of standard LED vehicle rear lights. The message to send is generated by a microcontroller board based on an ARM Cortex-M7 processor running at 600 MHz. The microcontroller commands the LED vehicle rear lights through an LED driver board isolated by an optocoupler. The VLC emitter center wavelength is 620 nm.

In line with the IEEE 802.15.7 standard, the VLC emitter prototype uses OOK modulation along with Manchester coding and enables data rates between 11 and 100 kb/s. As debated in the analyses presented in Section 2 and Section 3, the use of the light dimming mechanism based on the insertion of compensation symbols significantly downgrades the data rate. In such conditions, in order to efficiently implement the light dimming function, the proposed VLC emitter was modified for VPPM-based pulse-width control dimming method when the payload is transmitted. The high-performance microcontroller allows it to generate pulses in the microsecond range, which in turn facilitates a highly precise light intensity variation. Nevertheless, while a better resolution is possible with the algorithm proposed in IEEE 802.15.7, it should be mentioned that it is still not possible to have data transmission under a minimum percentage, allowed, for example, by the base low-resolution visibility patterns. Increasing the base resolution for a more precise dimming control significantly affects the memory requirements. For example, a 1% dimming resolution necessitates 100 intervals of high/low pulses for each logical bit, whereas a 0.1% resolution necessitates 10 times more intervals for the same logical bit. At the same time, in order to keep the data rate values, the processing power must be increased as well.

**Figure 6:**
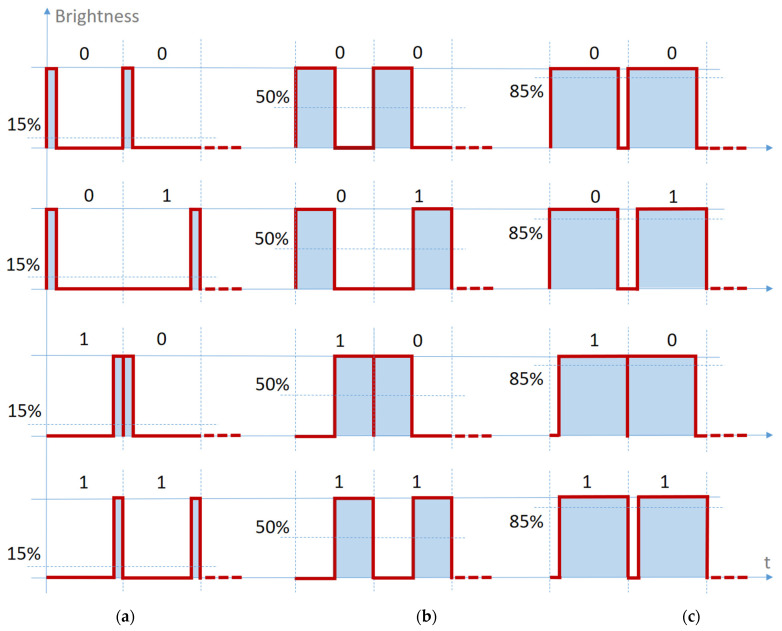
The VPPM standard scheme: (**a**) Average brightness less than 50%; (**b**) Average brightness equal to 50%, the same as Manchester coding; (**c**) Average brightness greater than 50%.

From our point of view, the VPPM method of dimming proposed by the standard IEEE 802.15.7 (Figure 6) can generate some issues in light dimming conditions, especially when a “1” bit is followed by a “0” bit. In such conditions, the two pulses join together, regardless of the duty cycle value, resulting in a single larger pulse. In limited noise conditions, combined with relatively high duty cycle values, the message extraction remains as simple as the measurement of the pulse width. However, in extreme light dimming conditions (e.g., 1%) corroborated with some potential low SNR values, it becomes difficult for the data processing unit to make the difference between a “*1*”-followed-by-a-”*0*” situation and a single “*1*” situation (Figure 6a). Thus, when such adverse conditions are fulfilled, the low SNR can alter the pulse width and give an erroneous result. The main advantage of the standard method is the possibility to control the duty cycle from 0% to 100% (of course, with no data transmission possibilities at the extremes).

**Figure 7:**
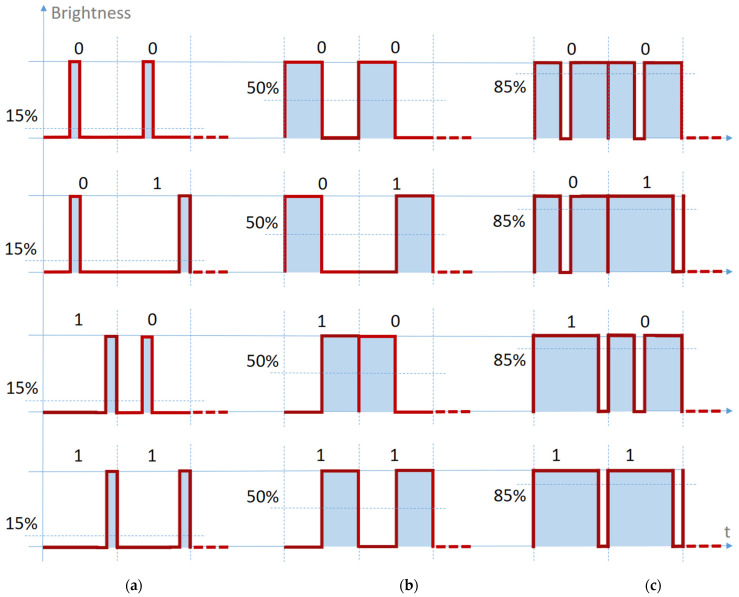
The scheme implemented in this work: (**a**) Average brightness less than 50%; (**b**) Average brightness equal to 50%, the same as Manchester coding; (**c**) Average brightness greater than 50%.

In light of the above, for enhanced resilience to noise and improved BER performances in light dimming conditions, the standard VPPM modulation has been adapted with an improved code, illustrated in Figure 7, and defined in Table 3. As seen in Figure 7a, the proposed VPPM method does not generate situations in which a dimmed message leads to the joining of two narrow-width pulses. Two pulses can only join in the case of a 50% pulse width (which replicates the Manchester code), as seen in Figure 7b, but due to the wider pulse width, this situation enables an adequate pulse-width measurement and a clear differentiation between the data bits. The main issue with this approach is the impossibility to increase the duty cycle over the 50% value. In order to overcome this impediment, the proposed code will switch the high and low levels between them for any duty cycle greater than 50%, as seen in Figure 7c. In this way, the advantages presented will be retained, with the only observation that the coding and decoding will be done with the same software subroutine, but considering the high as low and vice versa.

**Figure 8:**
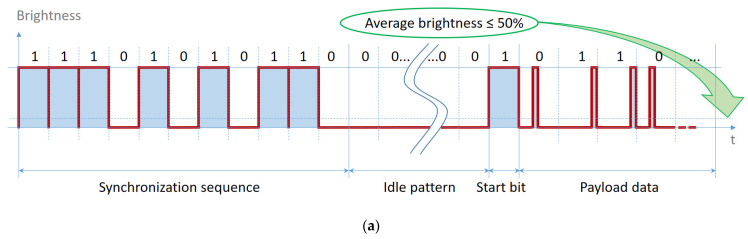

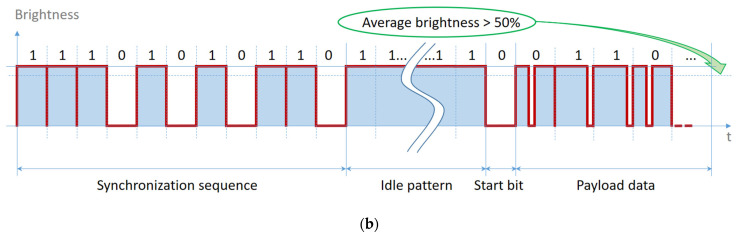
The structure of the proposed data frame: (**a**) Average brightness less than or equal to 50%; (**b**) Average brightness greater than 50%.

The structure of the proposed data frame is illustrated in Figure 8. The frame begins with a synchronization header, which informs the VLC receiver that a new data frame is being received. In order to provide a high frame delivery ratio, the synchronization header is transmitted with a fixed pulse width, as opposed to the payload transmission’s case. However, the dimming factor must be maintained; therefore, only for the synchronization header, the mechanism based on the insertion of compensation symbols is used. The duration of the idle period is dynamically determined, based on the duty cycle value calculated for payload, to attain a certain light intensity. The main software program will determine the idle pattern needed, which will be inserted after the synchronization header, followed by a start bit, which informs the VLC receiver that the data message is about to begin. The frame also contains a byte that transmits the length of the data frame to the VLC receiver. Finally, the frame contains a variable-length data message, followed by a short tail bits’ sequence that separates different data frames. Special care must be taken in determining the idle pattern, in order to prevent any light flickering. Therefore, it is mandatory to limit the light intensity fluctuations through the Maximum Flickering-Time Period (MFTP). The proposed data frame has a short synchronization sequence, which ensures the whole range of dimming with an idle pattern only formed with ones or zeros, as seen in Figure 8. Therefore, if the average brightness needed is less than or equal to 50%, the main software program will determine the low-level compensation time needed, which will be inserted after the synchronization header, followed by a high-level start bit, as seen in Figure 8a. If the average brightness needed is greater than 50%, the number of bits calculated and inserted will have a high-level value, and these will be followed by a low-level start bit, which precedes the payload, as seen in Figure 8b.

To further detail the light flickering aspects, it should be remembered that according to existing research, and most importantly according to the IEEE 802.15.7 standard, the human eye does not respond to light intensity modifications unless their frequency is higher than 100 Hz. To address this issue, the standard introduces a 5 ms MFTP. Basically, each MFTP should have the same light intensity, and thus, flickering is prevented. In practice, intra-frame flickering is prevented by using Run Length Limited (RLL) coding techniques, which ensure the same light intensity for bit 1 as for bit 0. Thus, all coding techniques specified in the IEEE 802.15.7 standard prevent flickering in this manner. The same is the case for the coding technique adopted in this article. Hence, to get back to the topic of this work, light dimming should not affect or disturb human eye, as long as there are no light intensity fluctuations (inside the data frame or between immediate frames), which is quarantined by the fact that no matter the duty cycle, the adopted coding techniques ensure the same light intensity for bits 1 and for bits 0.

### 4.2. Discussion of the VLC Receiver Structure

The proposed VLC receiver consists of three main stages, each with its own component blocks. The front stage is responsible for the optical signal collection and primary conditioning of the optical signal, the signal conditioning stage is responsible for the analog signal treatment and the digital signal regeneration, whereas the data processing stage is responsible for the analysis of the reconstructed signal, message decoding, and data extracting.

The optical front end is mainly based on a commercial PDA100A silicon optical detector. The optical detector consists of a PIN photodiode connected in a transimpedance circuit which transforms the incident optical signal into a proportional voltage. Since outdoor applications involve a multitude of optical noise sources, the design of the front stage becomes very important. Therefore, to improve the SNR, the front end uses a 2-inch optical lens, which focuses the incident light onto the optical receiver’s photosensitive surface. The optical lens reduces the VLC receiver’s FOV to ±20°, diminishing the effect of the parasitic light sources. Additionally, to further enhance the SNR, the VLC receiver englobes a band-pass optical filter, which only allows the passage of the wavelengths of interest. In this case, as the VLC emitter is based on a set of red rear lights, the optical filter only allows the passage of 600–680 nm signals. The optical front end based on this approach significantly improves the SNR, enhancing the results.

The front end has the purpose of conditioning the optical signal and transforming it into an electrical signal. From this point on, the electrical signal is being processed by the signal conditioning stage. At this level, the signal is filtered, gradually amplified from a few millivolts level to a 3.3 V level, and regenerated to its original square shape. The high-pass filter eliminates the DC component introduced by unmodulated light sources, such as the sun or unmodulated LED sources, as well as the low-frequency spectral components introduced by fluorescent and incandescent light sources, which introduce strong 100 Hz components. The low-pass filter eliminates the high-frequency noise components mainly represented by shot noise and thermal noise. To achieve this, a second order high-pass Bessel filter with a cut-off frequency of 500 Hz and a low-pass fourth order Bessel filter with a cut-off frequency of 500 kHz were used. This stage also englobes an automatic gain control stage which enables the VLC receiver to tolerate the variation of the incident optical power, caused by variable emitter–receiver distances or by different signal propagation conditions.

After that, the regenerated signal is processed in real-time by a 1008 MHz microcontroller board. Based on the length of the compensation time and knowing the structure of the synchronization header, the VLC receiver is able to determine the duty cycle, in order to correctly decode the received signal. At this stage, the data are decoded based on rising and falling edge identification and on pulse-width measurement. The high frequency of the microprocessor allows a high-resolution pulse-width measurement and adequate data decoding performances.

## 5. Experimental Results

### 5.1. Experimental Procedure and Methods

**Figure 9:**
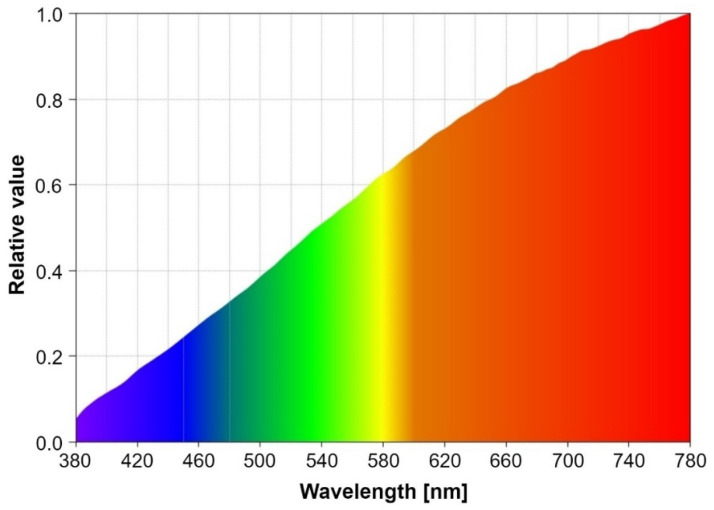
Spectral analysis of the incandescent light noise source used during the tests.

The objective of the experimental procedure is to evaluate the effect of light dimming on the performances of a vehicular VLC system. As already discussed in Section 4, the same software subroutine is used for duty cycle values greater than 50%, as well as for duty cycle values less than 50%. As such, the experiments carried out were done only for light dimming less than 50%, the results being valid for the other approach as well. For this purpose, two different setups have been chosen: an indoor setup and an outdoor setup. For each situation, the V2V VLC prototype has been tested for variable distances starting from 1 m and a duty cycle of 40%. From this point, the distance has been gradually increased until a maximum communications distance was achieved while maintaining a BER lower than 10^−4^–10^−3^. At each point, the BER has been determined in real-time, without using any error-correcting protocol. After that, the duty cycle has been gradually decreased from 40% to 1% and the measurements were repeated for each of these steps. For the indoor setup, the communication distance was restricted by the limited length of the laboratory (i.e., 40 m). In this case, the purpose was to evaluate the system in controlled conditions, in order to have a clear view concerning the light dimming effects. Thus, the indoor tests have been performed in high SNR conditions, with limited effect from natural daylight coming through the windows, and in low SNR conditions with an incandescent noise source consisting of six bulbs of 70 W each. At maximum power, this noise source can introduce an optical noise of up to 28,000 µW/cm^2^. Figure 9 presents the spectral analysis of the incandescent light source used to test the system’s noise resilience. One can see from this analysis that the incandescent noise source has its peak in the red region which is the spectral region for which PIN photodiodes have the highest sensitivity.

**Figure 10:**
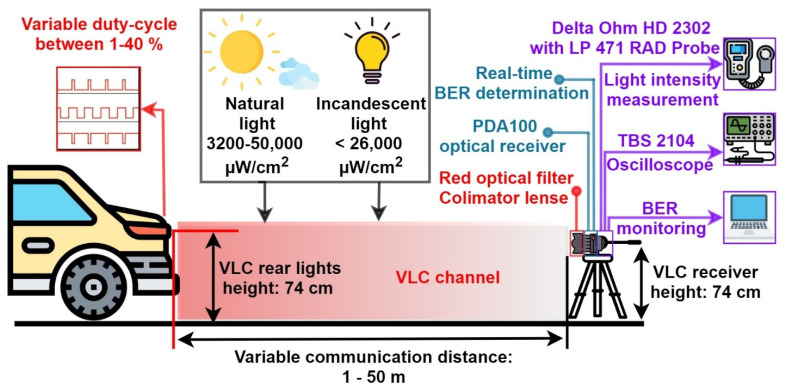
Schematic of the experimental setup.

The outdoor tests have been performed in uncontrolled daylight conditions on a mostly sunny day. In this case, the range could be increased up to 50 m, while the sun generated a variable noise component which reached values between 3200 and 50,000 µW/cm^2^ at the VLC receiver level. The summary of the experimental setup, the parameters of the parasitic light source, and the materials used during the experimental evaluation are presented in Table 4, Table 5 and Table 6, whereas Figure 10 illustrates the experimental methodology.

### 5.2. Experimental Determinations Concerning the Effect of Pulse-Width Variation on the Light Intensity Output

**Figure 11:**
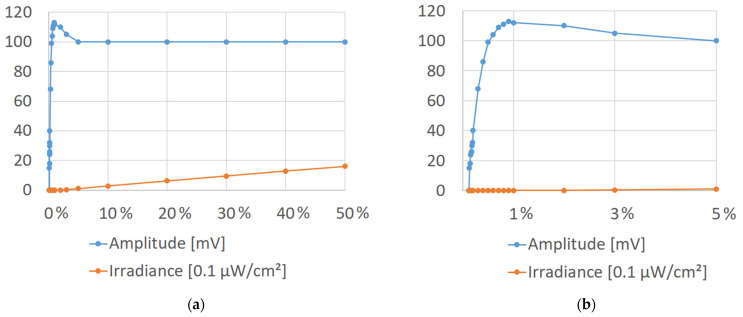
The effect of the pulse-width variation at the receiver level: (**a**) The light dimming effect; (**b**) The “lights-off” zone.

In order to understand the effect of pulse-width variation at the VLC receiver level, the emitter was positioned at 2 m distance, and a square signal with a frequency of 10 kHz was transmitted. The irradiance and the amplitude at the optical receiver output were measured, decreasing the duty cycle from 50% to 0.1%, with the PDA100A optical detector gain adjusted for 20 dB. The representation depicted in Figure 11a shows that the irradiance is directly proportional with the duty cycle rate until around 2%, when the intensity of light is too low to be detected by the irradiance meter (i.e., under 0.1 µW/cm^2^). On the other hand, the amplitude of the received signal is stable at around 100 mV until the duty cycle reaches 3%. Because the PDA100A has a bandwidth of 800 kHz when the gain is adjusted for 20 dB, at low duty cycle values, the signal starts to lose its squareness, appearing the effect of pulse overshoot. As such, the amplitude of the deformed pulse starts to increase until the duty cycle is lowered to around 1%, as seen in Figure 11b. Continuing to decrease the duty cycle, the pulse starts to drop in amplitude until the duty cycle reaches around 0.12%, when the LED lights of the emitter were unable to keep up with the fast-switching rate. Taking all this into consideration, at low duty cycle values, it is expected to see an impact on the maximum distance as well. Additionally, one can see that depending on the environment illuminance, with a duty cycle below 1%, the results can be considered as “lights-off.” It should be specified that this represents a particular case of light dimming in which the lamps are perceived as “off,” while the communication is still going on with ultra-short pulses. However, because the processing power necessary to decode a message transmitted with a duty cycle below 1% is too demanding, this proof of concept will be done with experiments in the 1% to 40% range.

### 5.3. Experimental Determinations Concerning the Effect of Pulse-Width Variation on Communication Performances

This section presents the experimental results showing the effect of the light dimming on the VLC link BER performances for indoor and outdoor setups.

#### 5.3.1. Indoor Testing Scenario and Results

The indoor experimental evaluation followed the path described in Section 5.1 and illustrated in Figure 10. Table 7 resumes the experimental results for the indoor evaluation. As one can see, the experimental results show that the VLC prototype is able to sustain a communication range of up to 40 m (restricted due to the length of the laboratory) even in light dimming conditions. Therefore, the VLC receiver is able to maintain a BER of 10^−6^ for distances between 1 and 40 m, while the duty cycle decreases from 40% to 1%. These results are extremely important, as they show that, in certain conditions, the VLC system is able to maintain high reliability while the distance is increasing and the duty cycle is decreasing to very low values.

**Figure 12:**
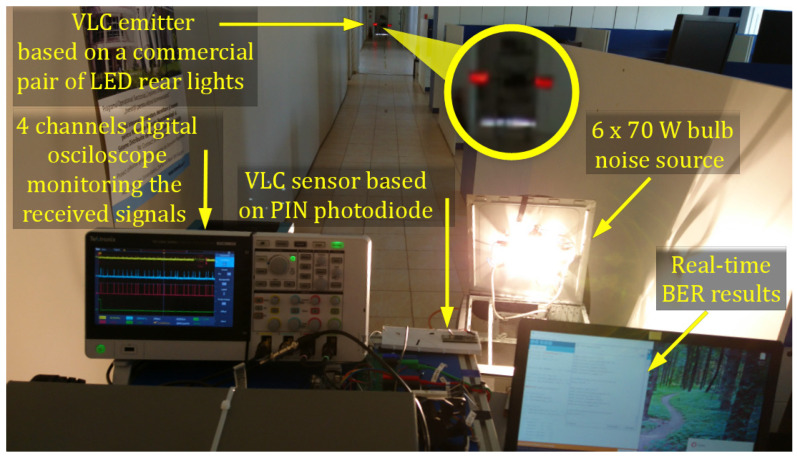
Indoor experimental setup.

As the BER results were not affected in any way by the duty cycle decrease, an optical noise source (see parameters in Table 5) has been used to depreciate the VLC channel. Therefore, the 420 W incandescent light source has been orientated toward the VLC receiver as illustrated in Figure 12. In such circumstances, when the VLC receiver is close to its maximum communication range and the amount of incident parasitic light pushes the VLC receiver close to its saturation limit, the number of bit errors significantly increases. Thus, one can see that in such an extreme case, a 1% duty cycle decrease (i.e., from 2% to 1%) increases the number of errors by two orders of magnitude. In these conditions, the 10^−6^ BER is maintained for a distance of up to 35 m, whereas after that, the BER has increased to around 10^−4^. This indicates that light dimming has a slightly negative effect on the SNR, and in turn, on the overall VLC performances.

**Figure 13:**
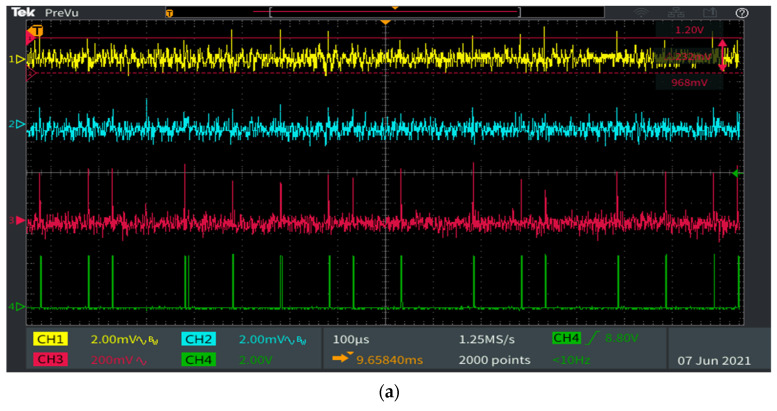

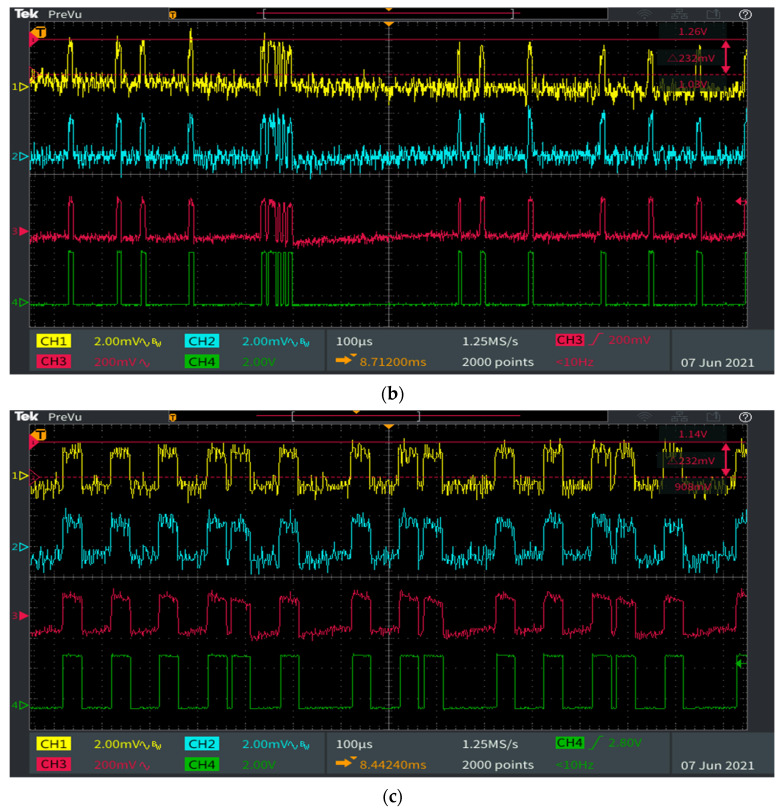
Oscilloscope print screens presenting the signal reconstruction process at the VLC receiver level: Channel 1 (yellow) contains the output of the optical receiver adjusted at 20 dB gain; Channel 2 (cyan) contains the output of the band-pass filter; Channel 3 (magenta) shows the output of the amplification blocks; Channel 4 (green) displays the reconstructed signal which is used for the data decoding process: (**a**) 1% duty cycle; (**b**) 10% duty cycle; (**c**) 40% duty cycle.

Figure 13 presents the signal reconstruction process at the VLC receiver level and it illustrates the impact of light dimming for a 40-m communication range. One can see that even if the SNR level is affected by parasitic light and by the increased communication distance, the adequate signal processing blocks are able to properly restore the data signal. This is possible due to an appropriate filtering mechanism, which enhances the SNR, due to the adaptive amplification, which compensates the attenuation caused by the increased distance, and due to the software decision algorithm, which enables proper data decoding even if the pulse-width recognition is slightly affected by the low SNR. All these mechanisms enable adequate data extraction even in unfriendly conditions.

#### 5.3.2. Outdoor Testing Scenario and Results

**Figure 14:**
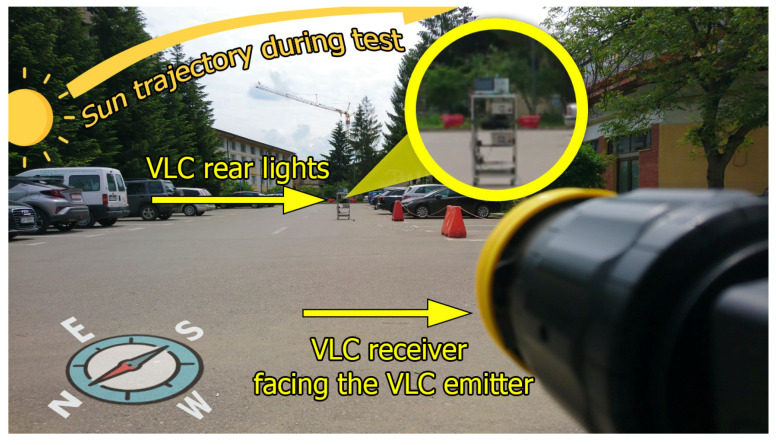
Outdoor experimental setup.

The outdoor experimental evaluation took place in the Stefan cel Mare University of Suceava parking lot, on a mostly sunny day, with clouds occasionally covering the sky. As suggested in Figure 14, the VLC emitter had a SE-NW orientation, whereas the VLC receiver was orientated toward the VLC emitter. In such circumstances, the VLC receiver is exposed to a strong and direct sunlight component, perturbing its functionality. Again, the outdoor testing scenario respected the methodology presented in Section 5.1. However, as the experiments were conducted in uncontrolled conditions, with the sun traveling the sky, changing the incidence angle of parasitic light, while the clouds were occasionally reducing its intensity, the test results were influenced by these factors.

**Figure 15:**
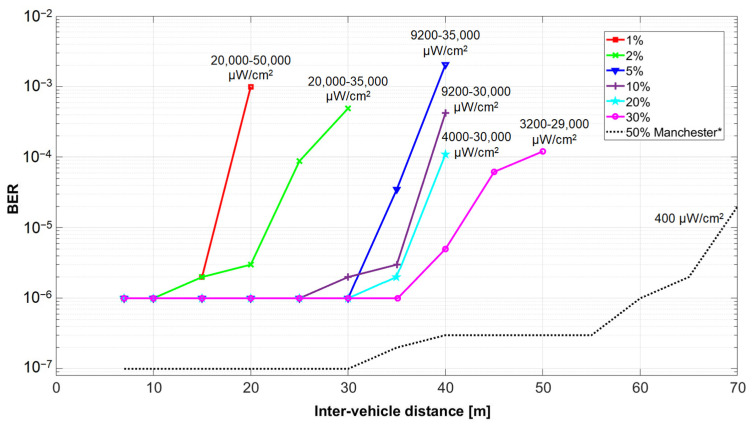
Summary of the V2V experimental BER results showing the influence of duty cycle reduction in outdoor sunny conditions. * The experimental results illustrating the 50% duty cycle have been introduced as a reference. These experiments have been performed in friendlier conditions, while using OOK modulation, Manchester coding, and a 100 kb/s data rate. Complete details concerning the experiments and the setup are available in [43].

The experimental results in outdoor conditions are summarized in Figure 15. In this case, the communication distance reached 50 m. These results suggest that in strong sunlight conditions, with parasitic irradiance at the VLC receiver level reaching 50,000 µW/cm^2^, the decrease of the duty cycle affects the link performances in terms of communication range, BER, and reliability. Thus, in such conditions, light dimming to 1–2% almost halves the communication range, while increasing the BER with three orders of magnitude. To have a clearer effect concerning the light dimming effect, Figure 15 includes for comparison purposes the results of a different experiment [43] performed without light dimming, on a less sunny day, with OOK modulation, Manchester coding, and a data rate of 100 kb/s. These comparative results show that the V2V VLC can provide a communication range of over 70 m. This comparison reconfirms the negative effect of light dimming on the SNR level.

Last but not least, it should be pointed out that although the ±20° VLC receiver FOV and the narrow-band optical filter significantly contributed to the SNR improvement, exposure to strong optical noise sources still affects the VLC link performances, especially in the cases when a low duty cycle is used.

## 6. Debate on the Results and on the Novelty of this Work

In the context of electric vehicles gradually replacing combustion engine vehicles, a very important challenge is to find solutions for more efficient energy management and for situations where the electric vehicles remain with critical battery charge. Therefore, this article is focused on the usage of light dimming on electric vehicles, while supporting V2V VLC. The envisioned scenario assumes that in extreme conditions, with almost no more energy remaining, or in situations where the lighting functions are less necessary (i.e., in daytime urban conditions), the electric vehicle can activate a light-dimming function, while also using the VLC technology to transmit its state to approaching vehicles.

In this context, although the effects of light dimming have been evaluated for indoor conditions in many studies, this is one of the very few articles which addresses this issue in middle-to-long-range automotive applications. For this purpose, a high-performance VLC system has been upgraded in order to evaluate the effect of light dimming on the reliability of a V2V VLC link. The experimental evaluation has been performed in controlled indoor conditions, and also in uncontrolled outdoor conditions.

The results showed that an optimally designed VLC system, and adequate front-end design, with a proper signal processing plan, the VLC receiver can have good performances even in light dimming conditions. Therefore, communication ranges up to 40 m can be maintained with a relatively low bit-error-rate. The experimental results indicate that in moderate-to-strong parasitic light conditions, the duty cycle decrease can be compensated with a proper signal processing plan. However, when the amount of parasitic light increases over a critical limit, or when the distance between the VLC emitter and the VLC receiver is close to the maximum coverage, the communication performances are affected. Thus, in such conditions, the communication range can be reduced to half, whereas the BER can increase by several orders of magnitude. These results confirm the findings of [22,44], which suggest that light dimming affects VLC performances. However, unlike in [44], where the communication distance reaches only 10 m, this new work provides a significantly higher communication range. It should be pointed out that the results presented in this work report communication ranges and noise robustness comparable to the results obtained by other VLC experiments without light dimming function. For example, [8,9,10,11,12,13,45] reported communication ranges that reached 50 m, while providing data rates between 10 and 100 kb/s. Along with all these works, this new article contributes to the demonstration of the high potential that VLC technology has in automotive applications. Thus, if a few years ago the reliability of VLC systems was rather questionable, whereas the communication ranges were rarely reaching 50 m, today’s prototypes are able to provide reliable communications in strong sunlight conditions [8,9], snowfall conditions [10], fog conditions [46], and mobile conditions [45], while providing low latencies [10,11,12] and high compatibility to 5.9 GHz RF-based communication solutions [2,3]. Now, with the results reported in this article, it has been demonstrated that such communication ranges could be achieved while also supporting light dimming. Thus, as far as we know, this article reports the longest vehicular VLC link achieved while providing light dimming down to 1%.

From a hardware design point of view, the implementation of the VLC prototype has shown that the light dimming function requires a significantly more powerful data processing unit. For example, in order to perform the light dimming function, the microcontroller performing data encapsulation at the VLC emitter level has been upgraded from 180 MHz to 600 MHz, whereas in order to be able to properly measure the width of the narrow pulses associated with the 1% dimming scenario, the microcontroller board of the VLC receiver has been upgraded from 180 MHz to 1008 MHz.

Another negative effect associated with light dimming is related to a significant data rate decrease. Therefore, the insertion of compensation symbols that decrease/increase the average lighting generates idle times when no data transfers are possible. Furthermore, when the processing power reach a certain limit, a higher resolution dimming can only be achieved by increasing the bit period, which means decreasing the data rate.

## 7. Conclusions

This article has provided an analysis concerning the manner in which automotive VLC systems could be used in light dimming conditions. This can be useful for improving road safety in a variety of scenarios. A broken-down car on the side of the road in the middle of the night could turn on the car’s lights-off VLC warning when the battery reaches the minimum allowed voltage instead of shutting down the lights completely.

Based on the experimental results provided by this article, one can consider that the use of VLC technology in V2V applications is highly promising, even in light-dimming conditions. The intensive evaluation showed that in low to high parasitic lighting conditions, an automotive VLC system can maintain a 10^−6^ BER even when the duty cycle decreases down to 1%. On the other hand, in cases of direct exposure to sunlight, a reduced duty cycle can affect the reliability of the VLC link, affecting the communication distance and increasing the BER.

Future work on this project will be focused on achieving higher resolution dimming and on evaluating the effects of lights-off communication on the V2V VLC link performances. Toward this aim, the duty cycle should be further decreased in order to be perceived by the human eye as being off. According to [32], LEDs appear to be off for the human eye when a duty cycle of 0.007% is achieved. Nevertheless, general-purpose high-power LEDs, such as the ones used in vehicle lighting systems, have switching times in the 0.1–1 µs range. Therefore, if the duty cycle is further reduced, the pulse width will be lower than the LED switching time. However, higher resolution dimming could still be achieved by further increasing the bit duration, which is equivalent to a data rate decrease. Nevertheless, further reducing the data rate below 10 kb/s seems inappropriate because such an approach would increase the message latency above the limits accepted by vehicle safety applications, so other modulation techniques must be taken into consideration for better results [15].

Another step of this project will be to find the optimal ratio for the duty cycle between maximum and minimum intensity of light, in a scenario where the driver is not braking, but the stop lights of the car still transmit the data with an intensity of modulated light dimmed at a level that can be considered as position lights by the current regulations.

## Figures and Tables

**Table 1:** PHY operating modes [16].

Modulation	RLL Code	Optical Clock Rate (kHz)	FEC		Data Rate (kbps)
			Outer Code (RS)	Inner Code (CC)	
OOK	Manchester	200	(15, 7)	1/4	11.67
			(15, 11)	1/3	24.44
			(15, 11)	2/3	48.89
			(15, 11)	none	73.3
			none	none	100
VPPM	4B6B	400	(15, 2)	none	35.56
			(15, 4)	none	71.11
			(15, 7)	none	124.4
			none	none	266.6

**Table 2:** Definition of data mapping for VPPM mode [16].

Logical Value	Physical Value *d* is the VPPM Duty Cycle *(0.1 ≤ d ≤ 0.9)*	
0	High	*0 ≤ t < dT*
	Low	*dT ≤ t < T*
1	Low	*0 ≤ t < (1−d)T*
	High	*(1−d)T ≤ t < T*

**Table 3:** Definition of data mapping for the proposed code.

Logical Value	Duty Cycle Value *d* is the Duty Cycle	Physical Value *T* Is the Bit Period	
0	1% ≤ *d* < 50%	Low	*0 ≤ t < (0.5d)T*
		High	*(0.5−d)T ≤ t < 0.5T*
		Low	*0.5T ≤ t < T*
	*d* = 50% Manchester	High	*0 ≤ t < dT*
		Low	*dT ≤ t < T*
	50% < *d* < 99%	High	*0 ≤ t < (d−0.5)T*
		Low	*(d−0.5)T ≤ t < 0.5T*
		High	*0.5T ≤ t < T*
1	1% ≤ *d* < 50%	Low	*0 ≤ t < (1−d)T*
		High	*(1−d)T ≤ t < T*
	*d* = 50% Manchester	Low	*0 ≤ t < (1−d)T*
		High	*(1−d)T ≤ t < T*
	50% < *d* < 99%	High	*0 ≤ t < (1−d)T*
		Low	*(1−d)T ≤ t < T*

**Table 4:** Summary of the experimental parameters.

Parameter	Feature/Values
Testing conditions	High SNR indoor conditions Indoor conditions In the presence of parasitic lights (low SNR) Outdoor, uncontrolled conditions
Dimming factor	1–40%
VLC emitter	LED-based vehicle rear lights
Emitter-Receiver (V2V) distance	1–50 m
VLC receiver	PIN Photodiode-based
VLC receiver height	74 cm
Modulation technique	VPPM
Data rate	10 kb/s
Measured parameter	Real-time BER determination without the use of forward error correcting protocols

**Table 5:** Summary of the light source parameters.

Parameter	Feature/Value
Light source	Incandescent light source
Light source power	6 × 70 W
Light color temperature	3254 K
Light source irradiance	Up to 28,000 µW/cm^2^

**Table 6:** Equipment used during the tests.

Equipment type	Equipment
Spectral analyzer	Sekonic C800
Irradiance meter	Delta Ohm HD 2302.0 with LP 471 RAD Probe
Oscilloscope	Tektronix TBS 2104

**Table 7:** Summary of the experimental results for the indoor tests.

Pulse Width	Modulation	Data Rate (kB/s)	VLC Distance (m)	BER	Conditions
1–40%	VPPM	10	1–40	<10^−6^	High SNR conditions: no artificial light sources and limited daylight
1–40%	VPPM	10	1–40	<10^−6^	Low SNR conditions: the VLC receiver is directly exposed to an incandescent light source of 18,500 µW/cm^2^
2–40%	VPPM	10	1–40	<10^−6^	Low SNR conditions: the VLC receiver is directly exposed to an incandescent light source of 26,000 µW/cm^2^
1%	VPPM	10	1–35	<10^−6^	
1%	VPPM	10	35–40	<1942 × 10^−4^	Low SNR conditions: 26,000 µW/cm^2^ incandescent light
